# Effects of 6-Month Square Stepping Exercise Intervention on Physical and Cognitive Competence, Regucalcin, and Body Composition in Older People: Study Protocol for a Randomised Control Trial

**DOI:** 10.3390/ijerph19053086

**Published:** 2022-03-06

**Authors:** Juan Manuel Franco-García, Jorge Carlos-Vivas, Damián Pereira-Payo, José Carmelo Adsuar, María Mendoza-Muñoz, Manuel Granado-Sánchez, Raquel Pastor-Cisneros, Laura Muñoz-Bermejo, Sabina Barrios-Fernández, Hadi Nobari, Narcis Gusi, Jorge Pérez-Gómez

**Affiliations:** 1 Health, Economy, Motricity and Education (HEME) Research Group, Faculty of Sport Sciences, University of Extremadura, 10003 Cáceres, Spain; jmfrancog@unex.es (J.M.F.-G.); dpereirab@alumnos.unex.es (D.P.-P.); hadi.nobari1@gmail.com (H.N.); jorgepg100@unex.es (J.P.-G.); 2 Promoting a Healthy Society (PHeSO) Research Group, Faculty of Sport Sciences, University of Extremadura, 10003 Cáceres, Spain; carmelo.adsuar@gmail.com (J.C.A.); mamendozam@unex.es (M.M.-M.); m_granado@unex.es (M.G.-S.); raquelpc@unex.es (R.P.-C.); 3 Social Impact and Innovation in Health (InHEALTH) Research Group, University Centre of Merida, University of Extremadura, 06800 Mérida, Spain; lauramunoz@unex.es (L.M.-B.); sabinabarrios@unex.es (S.B.-F.); 4 Physical Activity and Quality of Life Research Group (AFYCAV), Faculty of Sport Sciences, University of Extremadura, 10003 Cáceres, Spain; ngusi@unex.es

**Keywords:** dual tasks, elderly, fall-risk, haemoencephalography, SMP30, training

## Abstract

Background: Age-related changes negatively affect physical fitness, body composition, and executive function and produce a decrease in regucalcin level expression in blood. The square-stepping exercise (SSE) is a balance and lower-limb strength training programme used to prevent falls and stimulate cognitive function in older adults. This project aims to analyse the effects of SSE on executive function, regucalcin expression, fall prevention, body composition, and physical fitness in people over 65 years old. Methods: A randomized controlled trial will be conducted. A total of 90 older people over 65 years old will be recruited and randomly assigned to 2 groups: experimental (*n* = 45) and control (*n* = 45). The experimental group will perform an SSE-based intervention for 6 months (2 times per week), while the control group do not follow any treatment. Results: The main outcome will be balance, but other motor (body mass index, upper- and lower-limb strength, flexibility, and speed-agility) and cognitive variables (executive functions and attention) will be assessed. The expression of regucalcin levels will also be evaluated. Therefore, this project aims to analyse the effect of a 6-month SSE intervention on cognitive and motor competence, physical fitness, regucalcin levels, fall risk, and body composition in older people. If the intervention proves to be effective, it could be implemented in centres, entities, and associations specialized in elderly care.

## 1. Introduction

Ageing is associated with some body changes [1] such as decreases in muscle mass, bone mass, and cognitive function and increases in fat mass and blood pressure [2,3], which leads to worse functional health and, consequently, poorer quality of life. These changes can be aggravated by high rates of sedentary lifestyles, negatively affecting physical fitness, body composition, and executive function [4]. Thus, it is important to maintain the autonomy and independence of older people through “active” ageing, a term used by the World Health Organization (WHO, Geneva, Switzerland) that establishes physical exercise as an effective therapy to minimize and prevent the negative effects of ageing.

In order to know the negative effects of ageing, regucalcin has appeared as a calcium-binding protein discovered in 1978 [5] that has antitumor activity [6], is sub-expressed in cancerous tissues [7,8], and prevents apoptosis [9]. Although it presents numerous functions, its use as a biomarker that decreases with ageing in animals [7] and humans [10] is the special interest for the present project. Despite this, there is only one study [11] which analysed the effects of whole-body vibration physical exercise on regucalcin levels in humans, showing an increase in it.

As with regucalcin, ageing and a sedentary lifestyle also affects executive function. The study of executive function in humans has been approached using near-infrared haemoencephalography (nir-HEG). It is a novel technique that estimates brain activation in the frontal lobe from the amount of haemoglobin oxygenation in blood flow of this area [12]. To date, the few existing studies that use nir-HEG have been applied to children with mental problems or obesity and healthy people [13]. However, there is no available data related to executive function and falls in older people. Thus, the study of executive functioning could play an important role in the prevention of falls and performing motor activities and cognitive tasks simultaneously (known as Dual Task—DT) in older people [14].

Many of the tasks of daily life involve DTs, which are a matter of concern given that because of ageing, older people have problems performing the daily live activities simultaneously. The ability to perform any task may be impaired due to limited attentional resources [15], increased risk of falls [16], loss of independence, and impaired quality of life. Previous studies analysing the effect of DT in older women with fibromyalgia found that gait pattern is affected when an additional cognitive task is added to the primary motor activity; so the walking pattern may be safer and more stable if attention is focused on two simultaneous tasks [17,18]. However, no studies have investigated the impact of DT-based exercise programs on cognitive activity and executive function.

Square stepping exercise (SSE) is a type of physical exercise used in the elderly to reduce the risk of falls [19], but its effects on executive function and regucalcin levels have not been examined. Therefore, this study will analyse the effects of SSE on regucalcin, executive function, physical fitness, balance, fall risk, fear of falling, and body composition in older people. In addition, it will assess the relationship between changes in executive function and regucalcin, physical fitness, balance, fall risk, fear of falling, and body composition in older people.

We hypothesized that (1) SSE will enhance regucalcin levels, executive function, physical fitness, balance, fall risk, fear of falling, and body composition in older people, and (2) changes in executive function and regucalcin will be related to falls, body composition, and physical fitness in older people.

## 2. Material and Methods

### 2.1. Study Design

A randomized controlled trial with an assignment ratio of 1 to 1 to experimental and control groups will be conducted. The methodology followed will be the Consolidated Standards of Reporting Trials Statement (CONSORT) methodology [20].

### 2.2. Ethical Approval

The current protocol has been approved by the Bioethics and Biosafety Committee at the University of Extremadura (approval number: 109/2021) according to the Helsinki Declaration. Indeed, it has been registered in the Clinical Trials Registry provided by the Australian New Zealand Clinical Trial Registry (Request number: 383217; https://www.anzctr.org.au/, accessed on 29 November 2021).

### 2.3. Sample Size Calculation

The number of participants to be included in the studies has been calculated based on the change in plasma regucalcin levels, this actual minimum difference being 38.48 AU [21] with a power of 0.65. Hence, a total of 90 participants are required for the studies to be conducted (45 in the experimental group and 45 in the control group), accepting an alpha risk of 0.05. The common standard deviation in regucalcin is assumed to be 192.4 [11] and a loss-to-follow-up rate of 20% has been estimated.

### 2.4. Randomization and Blinding

Participants will be randomly assigned to the experimental (SSE training) or control groups. Prior to enrolling participants (1:1) the Research Randomizer software (version 4.0, Geoffrey C. Urbaniak and Scott Plous, Middletown, CT, USA; http://www.randomizer.org, accessed on 23 November 2021) [22] will be used in order to create a randomization sequence. A member of the research team with no active clinical involvement in the trial will conduct this process. Group assignment will be hidden in a password-protected computer file. Participants will be aware of their group assignment, but outcome assessors and data analysts will not know the participants group assignment.

### 2.5. Participants

**Figure 1:**
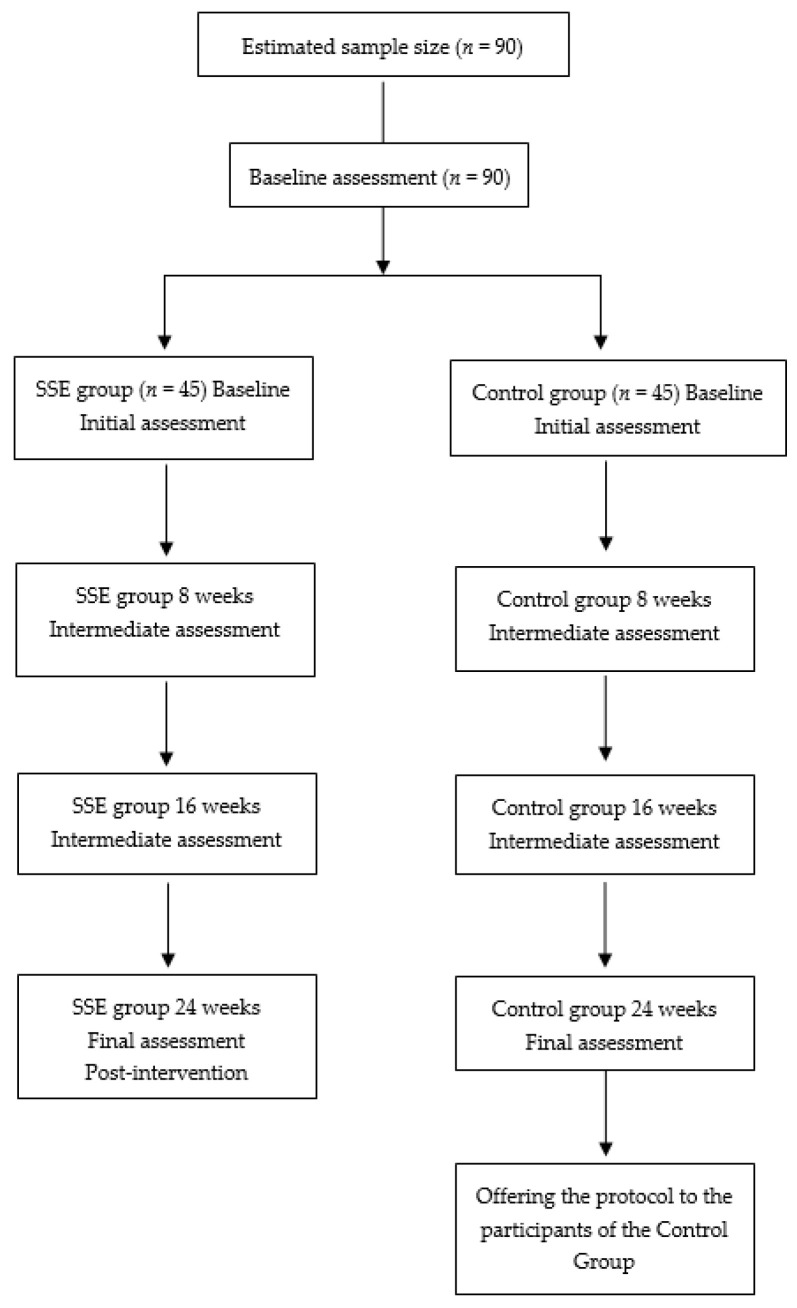
Study flowchart.

The participants will be recruited in Cáceres, Extremadura (West region of Spain). To be included in the study, participants will meet the following eligibility criteria: (a) age ≥65 years old; (b) not present or suffer any condition or inconvenience that prevents the normal practice of physical activity; (c) complete and sign the consent form; d) not engage in intense physical activity. The exclusion criterion is (a) presenting or suffering from any neurodegenerative pathology. The participants will be informed about the project via a phone call and meeting. All those who pass the inclusion criteria will be included in the study participation, as shown in Figure 1.

### 2.6. Intervention

#### 2.6.1. Experimental Group

The experimental group will perform the SSE twice a week during a total period of six months. At the start of the intervention, the trainer will state and clarify the training premises. A 200 × 100 cm-thin carpet, divided into 40 squares of 25 × 25 cm, will be used. Training sessions will consist of executing the SSE patterns separated into several mats at once. The SSE is composed by a total of 200 different movement patterns, classified as: beginner, intermediate and advanced [23]. The beginner level will consist of two sub-levels, while intermediate and advanced levels will both have three sub-levels. The intervention will progress through the first six levels up to the advanced level 3. Similarly, participants will start with simple movement patterns (e.g., two-step) and progress to increasingly complex patterns (larger steps per sequence). Table 1 shows the progression of SSE training that will be followed.

Table 2 shows the structure of an SSE training session in the experimental group. Before the beginning of each session, the instructor will show the guidelines to be followed. First, a general and specific warm-up will be performed. Then, the participants will proceed to learn and execute the patterns corresponding to that day; depending on their difficulty, the number of patterns executed will vary between three and five. Once finished, a cool-down consisting of stretching and brief relaxation will be performed to return the body to its initial state. At the end of each training session, a final thought will be conducted where participants will share their opinion on the patterns learned and will rate their perceived intensity of the work performed (from 0 to 10), taking into account the stress and fatigue that has been generated during the training session (Borg Scale) [24,25]. Participants should remain a rate of perceived exertion between 2 and 6.

#### 2.6.2. Control Group

Participators will continue with their typical lifestyle and routine. Once the experimental period is completed, the intervention will be offered to the control participants with no charge.

### 2.7. Outcomes and Procedures

Table 3 summarise several measures that will be taken to assess the utility and effectiveness of SSE program in the cognitive and motor domains (Table 3).

Data collection will take place on 2 days, at baseline, 2, 4 and 6 months, in identical order to avoid alterations. On the first day, blood will be drawn and body composition and risk of falls will be assessed, and on the second day, executive function and physical condition will be assessed. One of the researchers will oversee all measurements except for the blood draw (performed by a skilled worker).

#### 2.7.1. Primary Outcomes

- **Balance**. One leg-stance and four square steps test will be used to asses static and dynamic balance, respectively.
  ○ *One leg stance*. The test consists of standing on one leg for as long as possible (during a maximum lapse of 45 s) while keeping the arms crossed at all times. The test starts when the foot is lifted off the ground and ends when: (1) the arms are uncrossed, (2) the lifted foot touches the ground, (3) when more than 45 s have elapsed. Two variants of this test will be performed, with eyes open (ICC = 0.99) and with eyes closed (ICC = 0.95) [26,27]. Three repetitions of the test shall be performed, and the best time achieved will be recorded.
  ○ *Four square steps test (FSST)*. Four squares are formed by placing tape on the floor. At the start of the test, participants standing in the first square in front of square number 2 are asked to walk consecutively (1-2-3-4-4-4-3-2-1) without touching the holes and with both feet in contact with the ground. The time required to complete the full cycle will be recorded (ICC = 0.98) [28].
- **Applicability**. All the older people who can undertake the suggested training. If any participant is not capable to perform the intervention, the reason should be mentioned.
- **Safety**. In the event of any incident, damage, or trouble during the sessions, a record shall be kept and the source of the incident, injury, or problem noted.

#### 2.7.2. Secondary Outcomes

**Sociodemographic data**. Attendees will be questioned about their age, revenue, education level, and marital status.

**Height**. It will be assessed using a stadiometer (SECA 225, SECA, Hamburg, Germany). Subjects will stand with feet together, heels, buttocks, and upper back in contact with the scale and head in Frankfort’s plane.

**Bodyweight, fat mass and muscle mass**. Bodyweight shall be measured with a body composition analyser (TANITA MC-780 MA, Tanita Corp., Tokyo, Japan). Estimation of fat and muscle mass will be obtained by electrical bio-impedance using regression formulas.

**Executive function**. As part of the measurement of balance-related tests, subjects will be tested in DT using the nir-HEG headband to observe brain activation. The X-Wiz model will be used for cortical activation measurements in the Fp1 and Fpz/Cz regions of the Nir-HEG/Quantified-EEG/HRV system. The measurements will be based on the multimodal headband and pendant system developed to simultaneously combine the monitoring of Hemoencephalography in the frontal lobe (HEG), 2-channel Electroencephalography (EEG) by theta/Beta ratio, and heart rate (HRV) by the finger sensor [29].

**Regucalcin**. Blood samples will be obtained from the participants by fasting venous puncture in the antecubital vein (10 mL). It will use sterile material by qualified personnel, and these will be evaluated by Western blotting technique. The protocol has been described previously [11].

**Physical fitness**. They will be assessed with the following tests of the Senior Fitness Test Battery [30].

○ *The 6–min walking test.* This assessment determines the highest distance each participant can walk for 6 min around a 45.7 m rectangle [31]. This test has been shown to be valid and reliable (*R* = 0.94, CI = 0.90–0.96) [30].
○ *Lower-limb strength.* The 30-s Chair Stand Test involves of counting the total of times a participant can stand up entirely from a seated position with their back straight and feet parallel to the floor, not including pushing off with their arms inside 30 s [30] (*R* = 0.89, CI = 0.79–0.93).
○ *Upper-body strength*. Arm Curl test consists of lifting a weight as many times as possible (2.3 kg for women and 3.6 kg for men) by flexing and extending the arm for 30 s (*R* = 0.81, CI = 0.72–0.88). In addition, the handgrip strength test will be executed make use of a digital dynamometer (TKK 5101 Grip-D: Takey, Tokyo, Japan). The participants will perform the test two times (two tries per hand). The greatest value of the two efforts per hand will be taken, and the average of both hands will be treated for the study (ICC = 0.94–0.98) [32].
○ *Lower-limb Flexibility*. The Chair Sit and Reach Test will be applied (*R* = 0.95, CI = 0.92–0.97). Participants begin from a seated position with one leg stretched. Then, they slowly tilt forward by gliding their hands down the outstretched leg until they touch (or pass) their toes. The centimetres travelled from the toes will be recorded. One try will be performed for every leg and the average of both legs shall be used for analysis [33].
○ *Upper-limb Flexibility*. The Back-Scratch Test will be performed. Measures the motion range of the shoulder joint through the distance (or overlapping of) the middle fingers behind the back [34] (*R* = 0.96, CI = 0.94–0.98).
○ *Velocity*. The Brisk Walking Test will be employed. This test involves of determining the time needed by each participant to walk 30 m (ICC = 0.93) [35]. Two repetitions shall be performed with 1 min rest in between. The greatest outcome shall be considered for analysis.
○ *Agility*. The 8-Foot Up-and-Go Test will be applied. It consists of getting up from a chair, walking 8 steps and going around a cone to get back in the chair as quickly as possible (*R* = 0.95, CI = 0.92–0.97) [30].

**Physical Activity levels**. The International Physical Activity Questionnaire (IPAQ) short version will be applied [36]. This questionnaire provides information on the time spent walking, in vigorous and moderate-intensity activity and sedentary activity. The participants will be instructed to refer to all domains of physical activity.

**Fall Risk**. The 21-item Fall Risk Index (FRI-21) will be applied. It is a questionnaire comprising 21 items. Each item is given a rating of 1 (risk) or 0 (no risk), the amount of all items varies from 0 (minimal risk of falls) to 21 (extreme risk of falls). Elevated scores indicate a higher risk of falls. A cut-off point of 9–10 on the FRI-21 is useful for soon identification of falls risk (sensitivity 0.65) [37,38].

**Fear of falling**. The Falls Efficacy Scale-International (FES-I) will be used to measure fear of falling. It is a questionnaire created and authenticated by the Prevention of Falls Network Europe (ProFaNE). It has come to be a tool with excellent accuracy and authenticity for evaluating fear of falling (ICC = 0.96) in different societies and languages, including Spanish. [39,40,41]. The earliest questionnaire includes 16 items and is scored on a 4-point scale (1 = slightly worried to 4 = very worried). Therefore, the best value that can be obtained is 16 and the worst value is 64.

### 2.8. Statistics

Descriptive statistics and computations will be performed through SPSS (version 25.0; IBM SPSS Inc., Armonk, IL, USA). Data will be presented as means and standard deviation (SD).

Normality and homogeneity of data will be checked applying Kolmogorov–Smirnov and Levene’s tests, respectively. Moreover, an intention-to-treat and a per-protocol analyses will be executed:

**Intention-to-treat analysis**. This analysis will consider all randomly assigned participants in their respective group. Missing data will be input by multiple imputations. Repeated measures ANOVA will be applied to calculate the intervention effects on the different dependent variables, adjusted by age and baseline outcomes. Cohen’s d will be included in results (95% confidence interval) as the effect size. Statistical significance will be computed for the effect of time and the interaction group × time. Alpha level shall be set at *p* ≤ 0.05. Pearson’s bivariate correlations and linear regression will be also computed for analysing the associations between variables.

**Analysis by protocol**. For this analysis, only the volunteers who have completed more than 80% of training sessions will be considered. The same procedures as those indicated for intention-to-treat analysis will be conducted.

## 3. Discussion

Internationally, there are more than 300 publications on SSE in high-impact journals, but none have addressed the aspects that this project aims to study. There are many published studies on physical exercise in older people; however, there is no publication that studies the benefits of SSE on executive function, regucalcin, as well as studying the correlations of these changes with the variables of fall prevention, physical condition, and body composition in older people.

The implementation of the SSE has been investigated in adult populations [42,43], as well as in other populations with pathologies such as diabetes mellitus type 2 [44], Parkinson’s [45], and fibromyalgia [23], among others. Therefore, we hypothesize that a 6-month intervention based on SSE will improve the regucalcin levels, executive function, physical fitness, balance, fall risk, fear of falling, and body composition in older people over 65 years old. Furthermore, if the efficacy of this training system is demonstrated as an improvement in the regucalcin levels, it could reduce the risk of developing cancerous tissues, as well as being used as a biomarker in “active” ageing. Similarly, if there are encouraging results in cognitive performance and balance tests, it could indicate an improvement in functionality and performance in activities of daily living. These improvements will provide older people with independence from external aids that will be needed in the after-effects of ageing. Therefore, the implementation of SSE program could be applied by several agents interested in taking advantage of the benefits of this activity.

The SSE training method stands out because it requires neither expensive material nor any specific installation (it can be carried out in open and enclosed spaces) and reports a high applicability due to its adherence and feasibility [46]. Thus, it is a low-cost system standardized by different levels of difficulty (see methodology) that can be implemented at low cost in the public and private health sectors. Therefore, within the public health sector, the implementation of the SSE would be promoted through different services, such as the so-called “Exercise Looks After you” [47]. In the private sector, it is also possible to implement the SSE in associations or centres for the care or entertainment of the elderly.

This study protocol may have the following limitations: (1) difficulty recruiting individuals and having them adhere to a 24-week study plus the pre-intervention, intermediate intervention, and post-intervention assessment period; (2) the inability of some participants to complete the sessions due to COVID-19 infection; (3) there will be not follow-up phase after the end of intervention. Moreover, we recommend that future studies implement a follow-up phase at least one month after the end of the intervention. In addition, we encourage other researchers to replicate this research in adults or children to learn more about possible effects of SSE training protocols.

## 4. Conclusions

This project will investigate the effectiveness of SSE in people over 65 years of age for 6 months with the aim to analyse the effects of SSE on regucalcin, executive function, physical fitness, balance, fall risk, fear of falling, and body composition. Improvements in these variables will mean a reduced risk of developing cancerous tissues and improved performance in activities of daily living. If this intervention proves to be effective, it could be implemented in those centres, entities, and associations specialized in elderly care.

## Figures and Tables

**Table 1:** Square Stepping Exercise (SSE) progression for intervention.

Month	Weeks	Frequency (Days a Week)	Session Time (min)	Difficulty (Level)
1	1–2	2	40	Beginner 1
	3–4	2	40	Beginner 2
2	5–6	2	40	Intermediate 1
	7–8	2	40	Intermediate 2
3	9–10	2	40	Intermediate 3
	11–12	2	40	Advanced 1
4	13–14	2	40	Advanced 2
	15–16	2	40	Advanced 3
5	17–18	2	40	Advanced 3
	19–20	2	40	Advanced 3
6	21–22	2	40	Advanced 3
	23–24	2	40	Advanced 3

**Table 2:** Usual composition of an SSE session.

**Warm-up** (10 min)
Joint movement
**Main Part** (40 min)
Patch of the patterns realised in the prior session
Understanding and execution of SSE pattern 1
Understanding and execution of SSE pattern 2
Understanding and execution of SSE pattern 3
Understanding and execution of SSE pattern 4
**Cool-down** (10 min)
Stretch
Rest
Final thought

**Table 3:** Measurements calendar for both groups.

Assessment	Baseline	Month 2	Month 4	Month 6
Sociodemographic data	**x**			
Bodyweight	x	x	x	x
Height	x	x	x	x
Regucalcin	x	x	x	x
Executive function	x	x	x	x
Physical condition	x	x	x	x
Balance	x	x	x	x
Fall risk	x	x	x	x
Fear of falling	x	x	x	x

## Data Availability

Not applicable.
